# Routine testing for group B streptococcus in pregnancy: protocol for a UK cluster randomised trial (GBS3)

**DOI:** 10.1136/bmjopen-2024-087887

**Published:** 2025-06-17

**Authors:** Jane Daniels, Kate Walker, Lucy Bradshaw, Jon Dorling, Shalini Ojha, James Gray, Jim Thornton, Jane Plumb, Stavros Petrou, Jason Madan, Felix Achana, Susan Ayers, Georgie Constantinou, Eleanor J Mitchell, Soo Downe, Nicola Grace, Rachel Plachcinski, Tracey Cooper, Sarah Moore, Anne-Marie Jones, Eleanor Harrison, Joanne Brooks, Kerry Barker-Williams, Heidi Hollands, Sarah-Kate Mcleavey, Seren Willson, Sophie Webster, Jodi Carpenter, Meg Hyslop-Peart, Louise Wills, Rachel Haines, Rebecca Haydock, Shabina Sadiq, Linda Fiaschi, Lisa Evans, Reuben Ogollah, Jay Seale, Susanne Spas, Lixiao Huang, Sebastian Moody, Janine Abramson, GBS 3 Collaborative Group, Ihab Abbasi

**Affiliations:** 1 Nottingham Clinical Trials Unit, University of Nottingham, Nottingham, UK; 2 Centre for Perinatal Research (CePR), University of Nottingham, Nottingham, UK; 3 Neonatal Medicine, University Hospital Southampton NHS Foundation Trust, Southampton, UK; 4 Birmingham Women's and Children’s NHS Foundation Trust, Birmingham, UK; 5 Group B Strep support, Haywards Heath, UK; 6 Nuffield Department of Primary Health Care Sciences, University of Oxford, Oxford, UK; 7 Warwick Medical School, University of Warwick, Coventry, UK; 8 School of Health & Psychological Sciences, City St George’s University of London, London, UK; 9 School of Nursing and Midwifery, University of Central Lancashire, Preston, UK; 10 Grace Academy of Midwifery and Birth, Nottingham, UK; 11 National Childbirth Trust, London, UK; 12 Independent Parent and Public Involvement Consultant, Dewsbury, UK; 13 NHS England NW and Yorkshire, Leeds, UK; 14 School of Medicine, University of Nottingham, Nottingham, UK

**Keywords:** NEONATOLOGY, Randomized Controlled Trial, HEALTH ECONOMICS

## Abstract

**Introduction:**

It is unclear whether routine testing of women for group B streptococcus (GBS) colonisation either in late pregnancy or during labour reduces early-onset neonatal sepsis, compared with a risk factor-based strategy.

**Methods and analysis:**

Cluster randomised trial.

**Sites and participants:**

320 000 women from up to 80 hospital maternity units.

**Strategies:**

Sites will be randomised 1:1 to a routine testing strategy or the risk factor-based strategy, using a web-based minimisation algorithm. A second-level randomisation allocates routine testing sites to either antenatal enriched culture medium testing or intrapartum rapid testing. Intrapartum antibiotic prophylaxis will be offered if a test is positive for GBS, or if a maternal risk factor for early-onset GBS infection in her baby is identified before or during labour. Economic and acceptability evaluations will be embedded within the trial design.

**Outcomes:**

The primary outcome is all-cause early (<7 days of birth) neonatal sepsis, defined as either a positive blood/cerebrospinal fluid culture, early neonatal death from infection or a negative/unknown culture status with ≥3 agreed clinical signs or symptoms, who receive intravenous antibiotics ≥5 days. All women giving birth ≥24 weeks’ gestation, regardless of mode of birth, and all her babies will be included in the dataset. Cost-effectiveness will be expressed in terms of incremental cost per case of early neonatal sepsis avoided and incremental cost per quality-adjusted life-year associated with each strategy.

**Ethics and dissemination:**

The trial received a favourable opinion from Derby Research Ethics Committee on 16 September 2019 (19/EM/0253). The allocated testing strategy will be adopted as standard clinical practice by the site. Women in the routine testing sites will give verbal consent for the test. The trial will use routinely collected data retrieved from National Health Service databases, supplemented with limited participant-level collection of process outcomes. Individual written consent will not be sought. The trial results, and parallel economic, qualitative, implementation and methodological results, will be published in the journal Health Technology Assessment.

**Trial registration number:**

ISRCTN49639731.

**STRENGTHS AND LIMITATIONS OF THIS STUDY**

- This study is a cluster randomised trial to determine the clinical and cost effectiveness of routine testing for group B streptococcus in pregnancy.
- 320 000 participants will be adequately powered to detect a small but meaningful difference in early-onset neonatal sepsis.
- The study is not designed to detect a difference in early-onset neonatal sepsis between the two testing strategies but will provide information on acceptability and ease of implementation of the two testing strategies.

We use the term ‘women’ throughout to refer to those who are pregnant and give birth. We acknowledge that not all people who are pregnant and give birth identify as women, and it is important that evidence-based care for maternity, perinatal and postnatal health is inclusive.^1^

## Introduction

 One in four pregnant women in the UK carries group B streptococcus (GBS) in the gut and genital tract. Approximately 50% of babies whose mothers carry GBS will also be colonised, and of those, 3% will develop early-onset GBS (EOGBS) infection.^2^ EOGBS infection (occurring <7 days after birth) is caused by GBS bacteria ascending from the maternal genital tract during pregnancy (usually in the presence of ruptured membranes, although it can occur with intact membranes) or labour.^3^ EOGBS infections tend to manifest as pneumonia and sepsis and affect 1 per 1750 births in the UK (517 babies per year).^4^ GBS is the most common proven cause of early-onset infection (accounting for 40% of all isolates in culture positive cases) in the UK.^3^ One study has estimated that in the UK EOGBS infection causes more than 40 neonatal deaths and around 25 cases of long-term disability every year.^5^ Mortality is around 5%–10%, but higher among preterm babies (23%).^6 7^

Epidemiological studies have suggested that various factors (preterm labour, GBS colonisation or bacteriuria in the current pregnancy, a previous baby with GBS infection, maternal fever during labour) present at the time of birth are associated with the baby having an increased risk of developing GBS infection (presenting as either an early or late onset infection). However, a systematic review estimated that 65% of deliveries had no recognised maternal risk factors for GBS infection.^8^

Giving intrapartum antibiotic prophylaxis (IAP) to mothers who are known to carry GBS has been shown to reduce EOGBS infection.^9^ However, antibiotics may cause short-term complications for the mother (anaphylaxis, medicalisation of labour) or baby (effects on gut microbiome), may have long-term complications for the mother or baby^10^ and may increase antimicrobial resistance both for the individual and the wider population.

The current UK strategy involves identifying maternal risk factors for their baby developing EOGBS infection and then offering these ‘higher risk groups’ IAP.^11^ Additionally, women where GBS detected in a previous pregnancy are offered the option of bacteriological testing in late pregnancy and IAP if GBS-positive, or IAP without testing.

Universal testing for GBS is undertaken in many developed countries and has been attributed to the reduction in EOGBS infection. In the USA, the incidence of EOGBS infection per 1000 live births fell from 0.47 in 1999–2001 to 0.34 in 2003–2005 to 0.25 in 2010.^12^ The risk of EOGBS infection is significantly lower among infants of mothers undergoing universal testing than those who undergo a risk factor-based approach to prevention, with an adjusted relative risk (RR) of 0.46 (95% CI 0.36 to 0.60).^13^ The corresponding incidence in 2014–2015^4^ in the UK is 0.57/1000 births, a significant increase since previous surveillance undertaken in 2000 (0.48/1000)^7^ despite the introduction of a risk factor-based approach in 2003.^11^

The UK National Screening Committee (NSC) recommends ‘not to screen for maternal GBS carriage in the general population’ due to the absence of randomised trial data on either its effectiveness or cost-effectiveness.^14^ Testing would result in tens of thousands of women being offered and having IAP administered unnecessarily, while the long-term effects remain unknown. The key issue, when testing at 35–37 weeks of gestation was considered, was the lack of randomised trial data, evidence of efficacy and the accuracy of this antenatal testing as an indication of neonatal risk status at delivery.^15^ The NSC review recommended a randomised controlled trial, noting, however, that the positive predictive power of an antenatal testing policy for the outcome of a baby with EOGBS infection would be very low.

The enriched culture media (ECM) microbiological test is recognised as the international ‘gold standard’ for detecting GBS. It is highly sensitive, but maternal colonisation can alter through pregnancy.^16^ It takes at least 48 hours to produce a result and is usually offered at 35–37 weeks’ gestation, so misses most preterm births. Intrapartum rapid molecular tests have the potential to enable more accurately targeted IAP with current colonisation status, but the result may not be available in time for fast labours, there is no time for sensitivity testing for those allergic to penicillin, less time for the pregnant woman to be counselled and consider options if positive and it is more expensive than ECM.

### Trial objectives and design

#### Objectives

To determine whether routine testing of women for GBS colonisation either in late pregnancy or during labour reduces the occurrence of early-onset neonatal sepsis, compared with the current risk factor-based strategy.

To determine the cost-effectiveness, acceptability and ease of implementation of testing.

To compare two routine testing strategies (ECM and rapid test), to determine whether the proportion of women providing a sample for testing differs, whether either or both methods provide a timely result and the impact on neonatal admission.

#### Design

A multicentre prospective two-group parallel cluster randomised trial with embedded economic evaluation and qualitative study within a trial.

## Methods and analysis

### Setting and site level eligibility

Maternity hospitals (obstetric units (OU) and alongside midwifery units (AMUs)) if able to accept women requiring IAP are eligible to participate if, with training and support, they are able to implement the antenatal ECM or intrapartum rapid testing strategies. A list of 67 participating hospital trusts and health boards making up 71 sites in England and Wales is provided in online supplemental file 1.

One National Health Service (NHS) Trust may contain several maternity units. Each unit is then considered as an individual cluster site if the routine data sources could discriminate between the maternity units within the Trust and each maternity unit was able to maintain its allocated group.

The contracted microbiology laboratories providing services to the sites had to be prepared to use Public Health England (PHE, now UK Health Security Agency) Standard for Microbiological Investigations (SMI) B58 for the ECM testing for GBS for the duration of the trial. The PHE SMI B58 is the national guidance on the UK Standards for Microbiology Investigations for detection of carriage of GBS.^17^ Participating sites also had to be prepared to host a Cepheid GeneXpert system in a location convenient to the delivery suite.

Randomisation is at the site level to avoid any risk of contamination. Eligible sites are randomised on a 1:1 ratio to a routine testing strategy or to the risk factor-based strategy, using a minimisation algorithm with a random element.

Minimisation variables were overall number of births per year (<4000, 4000–4999 and ≥5000), according to national data for preceding year; neonatal unit level of care tier associated with the participating maternity unit (special care unit, local neonatal unit or neonatal intensive care unit)^18^ and presence of AMU.

There was a further second-level randomisation of the routine testing sites to either antepartum ECM testing or intrapartum rapid testing, restricted to achieve balance.

Withdrawal of sites after randomisation is avoided, if at all possible, in order to reduce bias.

### Individual-level eligibility

There are two levels of eligibility for individual women:

Testing level—eligibility to be offered an ECM or rapid test.

Dataset level—eligibility to be included in the dataset for analysis, regardless of whether test performed.

There is no exclusion based on the age of women or multiple births.

### Testing policy in each type of site

#### Inclusion criteria

- In ECM sites:
  - All women attending an antenatal clinic at ≥35 weeks of gestation without a planned delivery date.
  - 3–5 weeks prior to a planned induction date, or planned elective caesarean date prior to 40 weeks’ gestation.
- In rapid test sites:
  - All women who experience labour or prelabour rupture of membranes at ≥37 weeks’ gestation.
  - Women planning a home birth or in a free-standing midwifery unit (FMU) (which is not able to offer IAP) can be offered an antenatal rapid test which will be processed on the maternity unit/labour suite at ≥35 weeks gestation.
- In risk factor sites, all pregnant women at ≥24 weeks’ gestation. All women with risk factors should be reviewed for IAP/bacteriological testing in line with the Royal College of Obstetricians and Gynaecologists (RCOG) Greentop guidelines, detailed below.^11^

#### Exclusion criteria

- Women who do not provide verbal consent to have a swab.
- Women who have had a previous baby with GBS infection (early or late onset) and who want IAP. However, these women can still be offered a test and can choose to have IAP regardless of the result.
- Women in preterm labour (suspected, diagnosed, established), at ≤37 weeks gestation should be offered IAP routinely.
- In rapid test sites, women who have been admitted for a planned elective caesarean birth. (If women labour spontaneously at ≥37 weeks and plan not to proceed with the elective caesarean, they should then be offered a test.)
- In rapid test sites, women who require an emergency caesarean but who have intact membranes and are not in labour.
- Known congenital anomaly incompatible with survival at birth, of a singleton or all multiple fetuses.
- Known prelabour intrauterine death in the current pregnancy, of a singleton or all multiple fetuses.

Two categories of women should be offered IAP regardless of the result of the GBS test:

- Women who have previously had a baby with GBS disease should be offered a test but can choose to have IAP regardless of the result.
- Women with true GBS bacteriuria that has been treated with antibiotics earlier in the current pregnancy.

In all other cases, where the most recent antenatal ECM or intrapartum rapid test result differs from GBS status identified earlier in the pregnancy (such as detected as an incidental finding on a routine high or low vaginal swab), the later GBS status result should supersede the earlier one. The most recent GBS status should be used to determine the offer of, and administration of, IAP.

#### Dataset level criteria

In all units, all women giving birth ≥24 weeks’ gestation within their site’s study period, regardless of mode of birth, and all her babies will be included in the dataset unless they exercise their right to withdraw consent to use routine data, through the NHS data-opt out (or devolved nation equivalent).

Women who experience an intrapartum stillbirth will be included as they may have had testing for GBS, and GBS may be implicated in the aetiology of their stillbirth. If the stillbirth was antepartum, the woman will be included as she may have had antenatal testing for GBS prior to in utero death. However, women whose fetus (all fetuses in the case of multiples) had known congenital anomaly incompatible with survival at birth are excluded.

### Strategies to be compared

**Figure 1:**
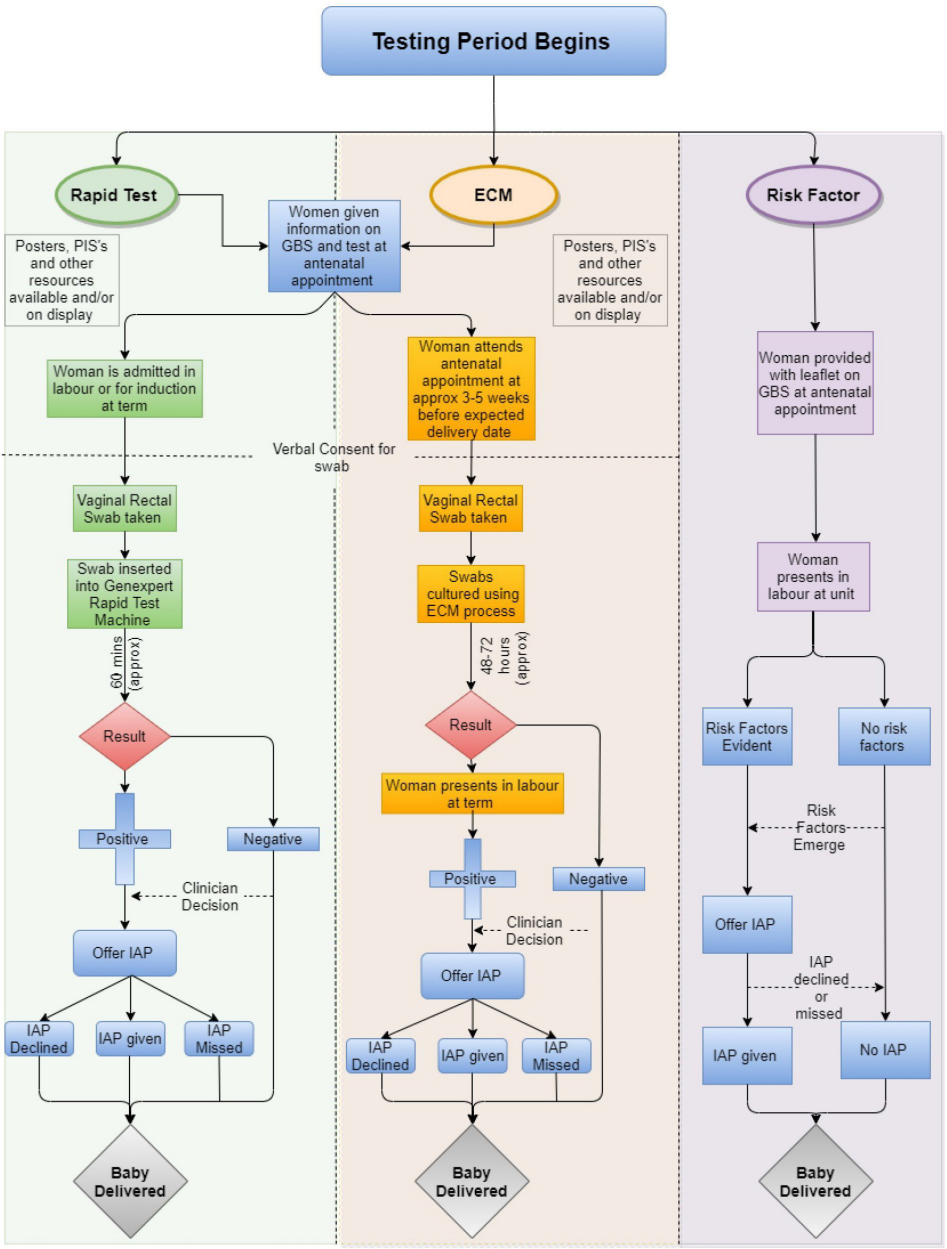
Overview flow chart of testing groups and risk factor groups. ECM, enriched culture media; GBS, group B streptococcus; IAP, intrapartum antibiotic prophylaxis; PIS, patient information sheet.

The routine testing strategies use antenatal ECM testing or intrapartum rapid testing using the Cepheid GeneXpert system, with IAP offered if the test is positive for GBS presence in the sample taken. The control strategy is to offer IAP if a maternal risk factor for EOGBS infection in her baby is identified before or during labour. Brief details of testing strategies are given in figure 1.

Blinding of women and healthcare professionals is not possible due to the nature of the strategies.

#### Antenatal ECM testing group

Sites randomised to ECM testing will obtain vaginal-rectal swabs from women at ≥35 weeks of gestation for those without a planned birth date (or 3–5 weeks prior to the planned birth date). If a vaginal-rectal swab is not collected in this time window, ECM testing should still be offered, providing a result can practically be achieved and communicated back to the clinical team and/or the woman in advance of the onset of labour.

The test is discussed with and offered to the woman. If she consents to testing, swabs will be obtained by a suitably trained member of the woman’s care team, or the woman may self-swab. The swab can be obtained at antenatal clinics, visits to hospital or in the community. All positive and negative results should be recorded and communicated to the women and clinical staff as per usual local procedures. If the results are not available when the woman goes into labour, risk factor-based guidance should be undertaken in regard to the offering and administration of IAP. The GBS3 ECM antenatal swab result should supersede any GBS result that has been obtained at an earlier gestation during the pregnancy.

The testing protocol is described in online supplemental appendix 2A. ECM testing will follow PHE Standards for Microbiology Investigation B58 and ID4(4) for GBS.^17 19^

#### Intrapartum rapid test sites

Sites randomised to rapid testing will collect vaginal-rectal swabs from women at ≥37 weeks’ gestation if they are in labour (latent or established) or about to be induced. The test is discussed with and offered to the woman. If she consents to testing, swabs are obtained by a suitably trained member of the woman’s care team, ideally before any vaginal examination (if one is to be performed). The testing protocol is described in online supplemental appendix 2B.

If the GBS test is positive, the woman should be informed and offered IAP. The swab should be offered up to the point of birth (sites were advised to use their clinical judgement, and if they did not feel they would have the results of the test in time to give IAP prior to birth, to revert to a risk factor-based approach).

#### Risk factor-based group (usual care)

Sites randomised to the risk factor-based screening and treatment approach will continue to use their current local guidelines which should be based on the RCOG Greentop Guideline 36.^11^ These state that women with the following risk factors for their baby developing EOGBS infection should be offered IAP:

Having a previous baby with GBS infection.

Discovery of maternal GBS carriage during pregnancy.

Preterm labour.

Suspected maternal intrapartum infection, including suspected chorioamnionitis.

Intrapartum fever.

Women who are known to have been colonised with GBS in a previous pregnancy should be offered the options of IAP or ECM testing in late pregnancy with the offer of IAP if GBS is detected. The risk of colonisation in subsequent pregnancies described in the RCOG guidelines should be discussed with the woman.

#### IAP and neonatal management

If a test is positive and the baby is born prior to the woman receiving IAP, neonatal management should be in line with National Institute for Health and Care Excellence (NICE)^20^ and the RCOG guidelines,^11^ covering both all cause and GBS specific infection. Monitoring of the infant and the decision whether to give neonatal antibiotics will be down to clinical judgement and the NICE guidelines.^20^

### Outcomes

#### Primary outcome

All-cause early neonatal sepsis is defined as starting at <7 days of birth. Cases will be identified from national data sources, a sample of which will be reviewed by a blinded adjudication panel (online supplemental appendix 3). Early neonatal sepsis is defined as:

- A positive culture of a pathogenic bacteria from blood or cerebrospinal fluid (CSF) taken at <7 days of birth.
- Death <7 days if infection or sepsis was recorded on the death certificate.
- Negative/unknown culture status with ≥3 agreed clinical signs or symptoms (see list below), for which intravenous antibiotics are given for ≥5 days, starting within 7 days of birth. If the infant was discharged or transferred prior to the completion of 5 days of intravenous antibiotics, the infant would still be classed as having sepsis if the intention was to treat for 5 or more days.

The following symptoms and signs are considered international standards for neonatal infection up to 28 days from birth and for babies born at all gestations and are level 2 of the Global Alignment on Immunisation safety Assessment in pregnancy consortium (GAIA case definition^21^) but with additional clarification derived from Modi *et al*’s case definition^22^ where the GAIA definition appeared ambiguous.

The following acute-onset clinical or laboratory features will be used as part of the definition of clinically suspected neonatal infection, if the blood and CSF cultures are negative/unknown and intravenous antibiotics are given for ≥5 days, starting within 7 days of birth:

- Increase in oxygen requirement or increase in ventilatory support or new or an increase in frequency of episodes of apnoea.
- Increase in frequency of episodes of bradycardia or hypotension (needing inotrope support or other intervention).
- Temperature ≥37.5°C or <35.5°C.
- Enteral feeds intolerance or abdominal distension.
*Reduced urine output to <1 mL/kg/hour.
*Impaired peripheral perfusion (capillary refill time >3 s or skin mottling or core-peripheral temperature gap >2°C).
- Irritability or lethargy or hypotonia (clinician-defined).
*Serum C reactive protein levels >15 mg/L or procalcitonin ≥2 mg/mL.
*White cells count 20×10^9^ cells/L or platelet count <100×10^9^/L.
- Glucose intolerance (blood) glucose (<2.2 mmol/L or >10 mmol/L) or metabolic acidosis (base excess <−10 mmol/L or lactate >2 mmol/L).

Note, signs or symptoms marked with an asterisk* may not be able to be identified from some routine sources.

An adjudication panel of UK consultant neonatologists will be convened to review the individual-level data of a sample of babies with clinically suspected sepsis. Two experts, masked to the location of birth and the neonatal unit, will review each case, state their individual opinion regarding the diagnosis of sepsis, and if not unanimous, a third expert will be involved to help reach a consensus. The adjudication panel will also review the individual-level data of babies who die during the intrapartum period, or when the timing of intrauterine death was unclear, to determine whether sepsis was the primary cause of death. Full details of the adjudication panel process will be included in a separate blinded endpoint Adjudication Committee Protocol (online supplemental appendix 3).

#### Secondary clinical outcomes

With the exception of maternal intrapartum anaphylaxis and systemic infection, all neonatal and maternal outcomes will be collected from routine data sources.

#### Neonatal

- Birth weight.
- Perinatal mortality (a stillbirth or early neonatal death, <7 days).
- Extended perinatal mortality (a stillbirth or neonatal death, <28 days).
- Baby death before discharge
- 5 min Apgar score.
- Fetal acidaemia, defined as cord arterial pH<7.05.
- Gestational age at birth.
- Admission for neonatal specialist care (length of stay, level of care).
- Seizures.
- Abnormal neurological signs (hypotonia or abnormal level of consciousness) at >24 hours of age.
- Late onset culture-positive (blood or CSF taken from 7 days to ≤28 days of birth) neonatal sepsis including clearly pathogenic organisms and excluding skin organisms (eg, coagulase-negative staphylococci).

#### Maternal

- Mode of onset of labour.
- Mode of birth.
- Duration of time from ruptured membranes to delivery.
- Duration of hospital stay.
- Change of intended location of childbirth.
- Maternal intrapartum anaphylaxis due to IAP.
- In a subset of participants for whom detailed data is collected, systemic infection confirmed with a positive blood culture or suspected maternal sepsis, from onset of labour to 42 days after birth.
- Maternal death, from onset of labour to within 42 days post partum.
- Cause of maternal death.

#### Process outcomes

It is important to collect and analyse process outcomes for usual practice and for both testing strategies, as failure to detect differences in early-onset sepsis may be due to poor compliance with the processes, rather than an intrinsic problem with the tests. It will also be important to measure any change in maternal IAP provision and/or neonatal care arising from the strategy allocated. These outcomes will be collected in a consecutive sample of at least 100 women per site, who give birth at gestational age ≥32 weeks (excluding women who have elective caesarean births at rapid test sites). Key parameters that will determine the feasibility and overall effectiveness of the risk factor-based strategy and the two testing groups will include:

- Number of women with risk factors for EOGBS infection developing in the baby and which risk factors they have.
- Number of women having a swab taken (of all those eligible for testing), including site (vaginal-rectal, vaginal only) and person performing the swab (self-swab, healthcare professional).
- Number of women who decline a swab when offered (and reasons why).
- Number of women who decline IAP when offered (and reasons why).
- Number of women having a swab taken at the appropriate time (of all those swabbed and all those eligible).
- Number of women with a test result available ≥2 hours and ≥4 hours before time of birth.
- Number of women receiving GBS-specific IAP, antibiotics for prophylaxis before operative (caesarean or instrumental) birth or intrapartum antibiotics for any other reason.
- Numbers of women with first dose of GBS-specific IAP administered at least 2 hours and at least 4 hours before birth.
- Total dose of administered IAP per woman.
- The proportion of women who tested positive for GBS, tested negative for GBS or who did not have an available test result.
- The proportion of failed tests.
- Of those who should have been offered IAP according to a positive test result or risk factors, the number of women offered IAP, and the number of women who were not offered IAP.
- Number of women with a negative test result or no documented risk factors who are offered and accept IAP (and reasons).

- Number of babies of mothers who
  - (A) Tested positive for GBS (testing sites).
  - (B) With documented risk factors (risk factor sites) whose vital signs and clinical condition were observed for at least 12 hours who were investigated for infection and/or had intravenous antibiotics commenced.

#### Additional descriptors

Descriptors of the dataset population as listed below will be collected and compared:

- Maternal age at booking.
- Parity at booking.
- Ethnicity.
- Smoking at booking.
- Index of Multiple Deprivation for maternal home at the time of childbirth.
- Number of fetuses (seen at dating ultrasound scan).
- Birth order.
- Baby’s sex.

The occurrence of an adverse event as a result of participation within this trial is not expected, and no adverse event data will be collected.

#### Trial phases

Testing sites have training and site-set up meetings with members of the trial team, including on-site training on the GeneXpert system if applicable. The testing strategies were implemented at sites for a period of up to 12 weeks before the start of the data collection phase.

During the 12 week implementation period, the number of tests performed as a percentage of those eligible for testing (from the site’s reported birth records) is monitored by the GBS3 coordinating centre. The target is at least 80% coverage (If actual birth rates are unavailable, estimation is based on previous years’ birth rate at site). Cascaded training by site staff trained on GBS3 during this implementation period should ensure all midwifery teams and midwives covering different shifts are trained promptly and appropriately. If the 80% testing coverage is met before 12 weeks, the site starts the study data collection period.

Sites will not be withdrawn if they fail to achieve the 80% test uptake rate by 12 weeks. At 12 weeks, the site will open to data collection regardless of testing coverage, and its data will be included in the primary analysis.

The first testing site entered the data collection period on 28 December 2021.

#### Detailed data collection of testing groups

For determining the process outcomes, individual-level data, not reported in the routine data sources, are required. To collect data on all women would negate the advantages of routine data use, so detailed data collection will be undertaken for a small subset of women at each site.

This will be retrospective source data collection using an online proforma designed for each testing strategy. At each site, individual data for a consecutive sample of at least 100 women per site, at gestational age ≥32 weeks, excluding women admitted for elective caesarean births, will be gathered. This will commence approximately halfway through the site’s data collection period.

This data will be extracted from the women’s healthcare records and transcribed by the research midwife at each site onto the GBS3-specific online database, using the NHS number, postcode and date of birth as the identifiers.

The detailed data collection will provide individual-level data associated with the testing coverage, IAP and resource use for approximately 4000 women in the risk factor-based usual care sites, 2000 women in ECM sites and 2000 women in the rapid testing sites.

#### End of data collection period

The GBS3 coordinating centre will inform the site that the standard data collection period at the site has ended and the site will revert back to the strategy undertaken prior to involvement in the GBS3 trial (eg, risk factor-based strategy). The site will be closed after completing all necessary close-out procedures, coordinated by the GBS3 coordinating centre.

The last site completed data collection on 31 March 2024.

### Routine data sources

Data sharing agreements between the sponsor and data provider will enable the University of Nottingham to receive routine data for the GBS3 Trial from various sources (table 1). Routine data and the detailed dataset will be safely stored in an accredited Trusted Research Environment (Data flow charts online supplemental appendices 4A–C).

**Table 1:** Routine data sources for GBS3 Trial

Data source	Description and use for GBS-3 trial
English Maternity Services Dataset (MSDS version 2) Maternity Services Data Set (MSDS) - NHS Digital and NHS Wales Informatics Service	These are patient-level datasets that capture key information at each stage of the maternity care pathway including the mother’s demographics, booking appointments, admissions and readmissions, screening tests, labour and delivery along with the baby’s demographics, admissions, diagnoses and screening tests. These will provide maternal secondary outcomes and other descriptive details.
UK Health Security Agency UK Health Security Agency - GOV.UK (www.gov.uk), Health Protection Wales Health Protection - Public Health Wales (nhs.wales)	Confirmed cases of all-cause early and late-onset neonatal sepsis and maternal sepsis identified through positive culture of a pathogenic bacteria.
National Neonatal Research Database Neonatal Data Analysis Unit | Faculty of Medicine | Imperial College London	Information on clinically suspected (negative or unknown culture status with ≥3 agreed clinical signs/symptoms, treated with antibiotics ≥5 days, within 7 days of birth) all-cause early neonatal sepsis, other secondary neonatal outcomes. All participating sites contribute data to this dataset.
Badgernet (Maternity)	Available as either a brief clinical summary record or a complete electronic health record system that captures all aspects of care and outcomes from booking to discharge from postnatal care. The latter system is used by more than 25% of English units. Data from the system would enable babies not ill enough to be admitted to a neonatal unit, and who remain on the postnatal ward, to be added to the trial dataset.
Badgernet (Neonatal)	This is the main source of the NNRD, along with additional variables which can add richer data for the primary outcome adjudication
Paediatric Intensive Care Audit Network Database	An audit database recording individual details of the diagnosis and treatment of all critically ill babies in paediatric intensive care units. This will identify newborns not admitted to the neonatal unit or discharged and later readmitted to hospital (within 7 days of birth) with sepsis
Hospital Episode Statistics (England) and NHS Wales Informatics Service.	These contain details of all admissions, outpatient appointments and accident and emergency attendances at NHS hospitals in England and Wales, respectively.
Office for National Statistics	This will capture neonatal deaths and maternal deaths that happen at home and were not reported to a hospital.

GBS, group B streptococcus; NHS, National Health Service.

### Sample size and justification

The sample size is based on the rate of all-cause early-onset neonatal sepsis between the routine testing and the risk factor-based randomised strategies. The NSC model^23^ estimated a rate of culture positive EOGBS as 0.49/1000 live births with the risk factor strategy. Assuming GBS contributes 50% of all early-onset neonatal infection,^24^ then the all-cause rate would be 0.98/1000 live births. The trial was designed to detect a 40% reduction in all-cause early-onset neonatal sepsis based on assuming that IAP would have an effect on 73% of early-onset neonatal infections caused by GBS and other Gram-positive infections, with no impact on the other 27% of cases caused by *Escherichia coli* and other Gram-negative infections and an effect of IAP on Gram-positive infections of 0.44, consistent with a Cochrane review^9^ and in line with the trends seen in the USA following introduction of testing.

To detect a 40% reduction (a reduction in event rate from 0.000986 to 0.0005916), with a 90% power and two-sided significance level of 5%, a total sample size of 2 12 960 women would be required without inflation for clustering effect. This infection rate estimate is conservative as it is based on culture-confirmed cases only, so the inclusion of clinically suspected cases will likely increase the power. There are no published estimates for the hospital-level intracluster correlation coefficient (ICC) for early-onset neonatal infection, but we would expect any variations in the infection rates across clusters to be a result of individuals’ clinical or demographic risk factors, biochemical or molecular markers, or bacterial load rather than hospital-level factors, hence we chose a small ICC of 0.0001. Assuming this ICC, an average cluster size of 4500 (calculated using published NHS Maternity statistics for deliveries in consultant-led or AMUs with a minimum of 3000 deliveries per annum) and allowing for a coefficient of variation in cluster size of 0.31 (CV), the design effect for the sample size would be around 1.5, calculated from the formula: Design effect=1+[(1+CV^2^) $\overset{-}{m}$−1]ρ where CV is the coefficient of variation, $\overset{-}{m}$ is the average cluster size, and ρ the ICC.

Adjusting for the design effect would lead to a total sample size of 320 000 women. These could be recruited from a minimum of 72 maternity sites.

During the course of the trial to date, we have reviewed the design effect parameters which were initially specified, to check any changes with time. We noticed that the average cluster size had reduced due to a reduction in birth rates since the original protocol and an increase in the NHS national data opt-out for use of data for research purposes in women of childbearing age in England. As a result, we opted to relax the requirement of a minimum of 3000 deliveries per year to 2000 deliveries per year (implemented 6 September 2022) to increase the pool of potential sites and, in addition, to allow variable data collection periods for each site from 9 to 16 months, rather than fixed at 12 months. Based on the number of births per year for participating sites, the projected length of the data collection period was determined such that by combining information on the average cluster size and CV, the effective sample size of 212 960 women would be maintained. This resulted in assuming an average cluster size of 4750 with a CV of 0.46.

### Statistical analysis

The analysis and reporting of the trial will follow the Consolidated Standards of Reporting Trials extension for cluster trials guidelines.^25^ The main comparative analysis of the trial will be according to the allocated group of the intended site of delivery of the baby (ie, intention-to-treat principle). All outcomes will be summarised by allocated group using frequency counts and percentages for categorical variables and means (and SD) or median (with lower and upper quartiles) for continuous variables.

A mixed effects logistic regression model will be used to compare the risk of early-onset all-cause neonatal sepsis in the testing sites relative to the usual practice sites, adjusting for the minimisation factors and accounting for the clustering effect due to sites and the correlation between outcomes for babies from a multiple pregnancy. Multiple births will be nested within site using random effects. All other minimisation factors will be adjusted for as fixed effects. The comparison will be presented as an adjusted risk ratio and risk difference with corresponding 95% CIs obtained using Stata’s margins command with SEs computed using the delta method. The maternity unit ICC with 95% CI will also be presented.

Sensitivity analyses for the primary outcome will include using cluster level analysis and multiple imputation for missing outcomes. Secondary analyses for the primary outcome will include excluding sites which failed to reach 80% uptake at the end of the implementation period, where the corresponding risk factor site opened at the same time as the routine testing site who failed to reach 80% testing coverage will be excluded, and to estimate the effect of each routine testing strategy compared with the risk factor-based strategy.

Subgroup analysis for the primary outcome will be conducted according to maternal ethnicity by including appropriate interaction terms in the analysis model. However, this analysis will be regarded as exploratory since the trial is not powered to detect interactions.

Between-group comparison of the secondary clinical (maternal and neonatal) and process outcomes will also be performed using mixed effect models appropriate for each outcome (linear for continuous outcome and logistic for binary outcomes), adjusting for the minimisation variables and the maternity sites as a random effect and, if applicable, will account for the correlation between outcomes for babies from a multiple pregnancy.

Full details of the planned analyses, including sensitivity and secondary analyses of the primary outcome, secondary outcomes and process outcomes, will be documented in the Statistical Analysis Plan prior to any analysis and made publicly available on the trial registry (ISRCTN49639731).

### Economic evaluation

We will conduct a decision-analytic modelling-based economic evaluation with the view to estimating the cost-effectiveness of alternative prevention strategies for GBS in pregnancy or labour, including intrapartum rapid testing, antenatal ECM testing and the current risk factor-based strategy.

The GBS3 trial will provide estimates of the incidence of early-onset all-cause neonatal sepsis as well as mortality and other morbidity outcomes. We will seek to match trial participant records to Hospital Episode Statistics (or devolved nation equivalent) and National Neonatal Research Database data in order to profile each trial participant’s duration and intensity of antenatal, intrapartum, postnatal and neonatal care, based on standard criteria for level of care, as well as maternal and neonatal surgical procedures and complications.^26^ In addition, targeted economic studies will be integrated into the GBS3 trial in order to generate key resource use and economic cost parameter estimates for the model. Specifically, the detailed data collection for 100 women within each trial centre, described above, will provide a vehicle for estimating resource use and cost profiles associated with antenatal ECM and intrapartum rapid testing, and IAP, as well as test and IAP uptake rates. Unit costs for each resource input will largely be derived from national secondary sources, for example, the Department of Health’s National Schedule of Reference Costs but supplemented where necessary using primary research methods and discussions with suppliers, for example, Cepheid.

The decision-analytical model will allow us to extrapolate the cost-effectiveness of alternative testing strategies for GBS colonisation in pregnancy beyond the parameters of the GBS3 trial. The model will consider the progression of early-onset neonatal sepsis over time, and the model structure will capture disease progression using health states that represent the important natural history and clinical-related and event-related activity for early neonatal sepsis, the appropriate model type (eg, Markov or discrete-event simulation approach) and the appropriate analytical framework (eg, cohort analysis vs individual-level simulation). Furthermore, the decision-analytic model will provide a framework for integrating data from external studies, for example, GBS1^27 28^ and GBS2.^29^ A key methodological challenge will involve generating expressions of cost-effectiveness amenable to broader cost-effectiveness comparisons by decision makers. Translating the potential benefits of alternative testing programmes in terms of episodes of early-onset neonatal sepsis avoided into quality-adjusted life-year (QALY) metrics is constrained by the paucity of validated utility measures in the perinatal and early childhood contexts.^30^ The utility values placed on health states within the model will be informed by our recent research in this area, which includes a systematic review of all published utility values for childhood health states.^31^ Multiparameter uncertainty in the model will be addressed using probabilistic sensitivity analysis.^32^ Cost-effectiveness acceptability curves will be used to show the probability of cost-effectiveness of each of the evaluated strategies at alternative cost-effectiveness thresholds held by decision-makers.^33^ Any costs occurring beyond the first year after birth will be discounted using nationally recommended discount rates.^34^

Economic outcomes will be expressed in terms of incremental cost per case of EOGBS avoided and incremental cost per QALY gained associated with alternative testing strategies for GBS in pregnancy or labour.

### Qualitative evaluation

We will conduct a qualitative study to assess the acceptability of routine GBS testing to women and health professionals, as well as determine the feasibility of implementing testing in different healthcare contexts. To do this, the study will recruit women and health professionals in four NHS sites randomised to either of the routine testing groups.

The aim of this qualitative study is to understand the acceptability of the different methods of routine testing for GBS colonisation to pregnant women and health professionals, identify the barriers and facilitators to the implementation of either routine testing strategy and understand how individual and site-level context and process mechanisms influence the acceptability of testing.

In-depth interviews via telephone or video call will be used to collect the data, and an interview topic guide informed by the theoretical framework of acceptability will be used. This framework focuses on affective attitudes, burden, perceived effectiveness, ethicality, intervention coherence, opportunity costs and self-efficacy.^35^ Site-specific contextual factors will be examined using the NICE guidelines on identifying barriers to changing practice which outline the practical, environmental and organisational barriers and facilitators to implementing changes in clinical practice.^36^

Purposive sampling of the participants will be employed to identify women from specific groups to take part, including variation in place of birth, preterm birth, age and ethnicity. Similarly, purposive sampling will be used with health professionals to ensure representation from midwifery, obstetric, neonatal and microbiology disciplines, with varied clinical experience across hospital (teaching and general), FMU/AMU and community settings. Written informed consent will be obtained before the interviews.

The final sample size for women and healthcare professionals will be determined by saturation within subgroups. It is anticipated that, to ensure adequate representation of different groups and saturation of themes specific to these groups, we will interview a minimum of 50 women. For healthcare professionals, we anticipate a minimum of 30 interviews will be needed to ensure adequate representation of different clinical disciplines and NHS services. More may be recruited if the data cannot yet be fully explained by the analysis after 30 interviews.

Interviews will be recorded and transcribed. Data will be analysed using thematic analysis^37^ and a framework method to provide a structured summary of the data including women’s and health professionals’ views on the acceptability of the different methods of routine testing, barriers and facilitators to implementation of either routine testing strategy and individual and site-level context and process mechanisms which may influence the acceptability of testing.

## Ethics and dissemination

The trial received a favourable opinion from Derby Research Ethics Committee (REC, reference 19/EM/0253, date 16 September 2019), Section 251 support from the Confidential Advisory Group (CAG), global approval from the Health Research Authority (HRA) and local Trust/ Board level approval. Protocol version 6 date 13 December 2023 is the prevailing version.

Pregnant women will be asked to consent to swab testing and treatment according to the policy to which the centre has been allocated. However, they will not be asked to consent to participate in the GBS3 cluster trial. This is intentional because individual consent within a cluster risks biased exclusion due to knowledge of the treatment group and leads to unreliable estimates of testing effectiveness.^38^

The comprehensive project results will be collated in the National Institute for Health Research (NIHR) Library under collaborative group authorship. The component studies (clinical trial, qualitative and economic) will be published together or individually in high-impact peer-reviewed journals and by presentation at medical and midwifery conferences locally, nationally and internationally. It is anticipated that there will be several secondary publications, addressing additional objectives or methodological observations beyond those described in this protocol. Publication of such secondary data will only be permitted before the main results if doing so will not jeopardise the integrity and interpretation of the main results.

Requests for data collected for the GBS3 trial from parties outside the Trial Management Group will be considered by the Nottingham Clinical Trials Unit (NCTU) Data Sharing review panel and will be subject to a data sharing agreement. Participant-level data from the routine data sources will not be available under the terms and conditions under which NCTU receives the data.

The babies born to women in the GBS3 trial create a unique population with detailed perinatal data. Long-term follow-up of this population can explore the association between perinatal factors, for example, intrapartum and postnatal exposure to antibiotics, the neonatal microbiome and childhood conditions such as asthma and inflammatory bowel disease. It will also be valuable to record the long-term sequelae of babies who have suspected or culture-confirmed early and late neonatal sepsis, including educational attainment. Approval will be sought to retain all data received from routine data providers, subject to further funding.

### Parent and public involvement

There has been detailed input into all aspects of the project from Group B Strep Support (www.gbss.org.uk), the UK charity working to stop GBS infections in babies, and NCT, the UK charity providing antenatal and postnatal information in support of birth and parenthood. Representatives from both are coinvestigators, and GBSS’ chief executive has led the PPI group.

Links to further information about GBS have been provided by Group B Strep Support, NCT and the RCOG and will be available on the trial website. Two closed Facebook groups have been set up, one for parents with lived experience of GBS infection in their baby and a more general maternity group. The Parent and Public Involvement (PPI) groups have reviewed and provided feedback on all public-facing information and information provided to healthcare practitioners and commented on the appropriateness of the use of routine data. At the end of the study, they will help in creating plain language summaries of the results and their dissemination to parent audiences.

GBSS and NCT have helped publicise the trial, via their websites, newsletters and social media feeds. GBSS has advocated for hospital level participation through its media and parliamentary contacts and is providing structured advice to expectant parents regarding the trial and testing strategies via its helpline. Both NCT and GBSS will be instrumental in the dissemination of the trial results and will update their own information resources with the results and the implications of GBS3.

### Trial steering and data monitoring committees

Trial steering committee (TSC) independent members: David Torgerson (Chair), Anna Curley, Androulla Efstratiou, Maeve Eogan, Rachel Roberts, Julia Sanders, Jan Van der Meulen.

Data monitoring committee (DMC) independent members: Stephen Walters (Chair), Ruth Gilbert, Ben Mol.

The TSC will meet (in person ideally) prior to commencement of the accrual and then at a minimum of once yearly (in person or remotely) and will provide independent oversight of the trial and associated studies on behalf of the trial sponsor.

The DMC will meet (in person ideally) prior to commencement of the accrual and then at a minimum of once yearly (in person or remotely) to independently assess safety, effectiveness and futility of the trial and will report to the TSC. Full details of both the TSC and DMC will be outlined in a charter.

## Supplementary material

10.1136/bmjopen-2024-087887 online supplemental file 1

10.1136/bmjopen-2024-087887 online supplemental file 2

10.1136/bmjopen-2024-087887 online supplemental file 3

10.1136/bmjopen-2024-087887 online supplemental file 4
