# Supplementary material for: Routine testing for group B streptococcus in pregnancy: protocol for a UK cluster randomised trial (GBS3)
Source: BMJ Open. 2025 Jun 17;15(6):e087887. doi: 10.1136/bmjopen-2024-087887 (PMC12182030; doi:10.1136/bmjopen-2024-087887)
Supplement: online supplemental file 1 [file bmjopen-15-6-s001.docx]

## Appendix 1. GBS3 participating sites

| Airedale NHS Foundation Trust |
| --- |
| Ashford & St Peters Hospitals NHS Foundation Trust |
| Barts Health NHS Trust |
| Bedfordshire Hospitals NHS Foundation Trust |
| Betsi Cadwaladr University Health Board |
| Birmingham Women's and Children's NHS Foundation Trust |
| Blackpool Teaching Hospitals NHS Foundation Trust |
| Bolton NHS Foundation Trust |
| Bradford Teaching Hospitals NHS Foundation Trust |
| Calderdale and Huddersfield NHS Foundation Trust |
| Cardiff and Vale University Health Board |
| Chelsea and Westminster Hospital NHS Foundation Trust |
| Chesterfield Royal Hospital NHS Foundation Trust |
| Countess of Chester NHS Trust |
| County Durham and Darlington NHS Foundation Trust |
| Doncaster and Bassetlaw Teaching Hospitals NHS Foundation Trust |
| East and North Hertfordshire NHS Trust |
| East Lancashire Hospitals NHS Trust |
| East Suffolk and North Essex NHS Foundation Trust |
| Epsom and St Helier University Hospitals NHS Trust |
| Frimley Health NHS Foundation Trust |
| Guys and St Thomas NHS Foundation Trust |
| Homerton University Hospitals NHS Foundation Trust |
| Hywel Dda University Health Board |
| Imperial College Healthcare NHS Trust |
| Kettering General Hospital NHS Foundation Trust |
| Kings College Hospital NHS Foundation Trust |
| Lancashire Teaching Hospitals NHS Trust |
| Leeds Teaching Hospitals NHS Trust |
| Lewisham and Greenwich NHS Trust |
| London North West University Healthcare NHS Trust |
| Maidstone and Tunbridge Wells NHS Trust |
| Manchester University NHS Foundation Trust |
| Medway NHS Foundation Trust |
| Mersey and West Lancashire Teaching Hospitals NHS Trust (formerly St Helens and Knowsley NHS Trust Teaching Hospitals and Southport and Ormskirk Hospital NHS Trust) |
| Mid Cheshire Hospitals NHS Foundation Trust |
| Milton Keynes Hospital NHS Trust |
| North Tees and Hartlepool NHS Foundation Trust |
| North West Anglia NHS Foundation Trust |
| Northern Care Alliance NHS Foundation Trust |
| Northumbria Healthcare Foundation Trust |
| Nottingham University Hospitals NHS Trust |
| Royal Berkshire NHS Foundation Trust |
| Royal Devon and Exeter NHS Foundation Trust |
| Royal Free London NHS Foundation Trust |
| Royal United Hospitals Bath NHS Foundation Trust |
| Sherwood Forest Hospitals NHS Foundation Trust |
| South Tees Hospitals NHS Foundation Trust |
| South Tyneside & Sunderland NHS Foundation Trust |
| South Warwickshire University Foundation Trust |
| Mid and South Essex NHS Foundation Trust |
| St Georges University Hospitals NHS Foundation Trust |
| Stockport NHS Foundation Trust |
| Surrey and Sussex Healthcare NHS Trust |
| The Mid Yorkshire Hospitals NHS Trust |
| The Newcastle upon Tyne Hospitals NHS |
| The Princess Alexandra Hospital NHS Trust |
| University Hospitals Birmingham NHS Foundation Trust |
| University Hospitals Coventry and Warwickshire NHS Trust |
| University Hospitals of Derby and Burton NHS Foundation Trust |
| University Hospitals of Leicester NHS Trust |
| University Hospitals Plymouth NHS Trust |
| University Hospitals Sussex NHS Foundation Trust |
| Warrington and Halton Teaching Hospitals NHS Foundation Trust |
| West Suffolk Hospital Foundation Trust |
| Whittington Health NHS Trust |
| Worcestershire Acute Hospitals NHS |
