# Supplementary figures and images for: Routine testing for group B streptococcus in pregnancy: protocol for a UK cluster randomised trial (GBS3)
Source: BMJ Open. 2025 Jun 17;15(6):e087887. doi: 10.1136/bmjopen-2024-087887 (PMC12182030; doi:10.1136/bmjopen-2024-087887)

##

## Appendix 2. Testing protocols

2a. ECM testing protocol


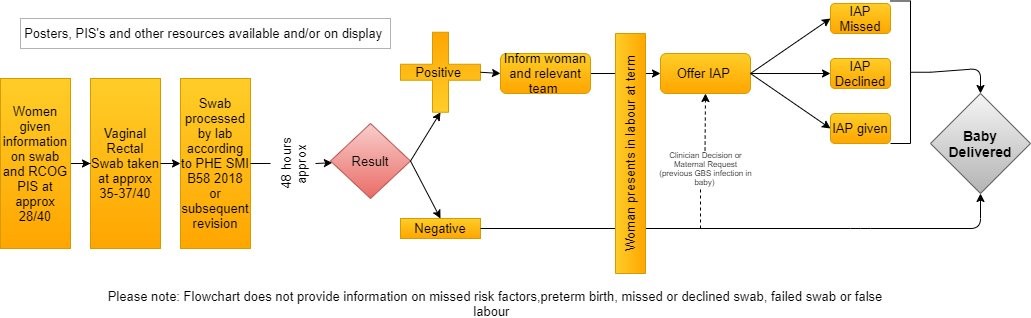


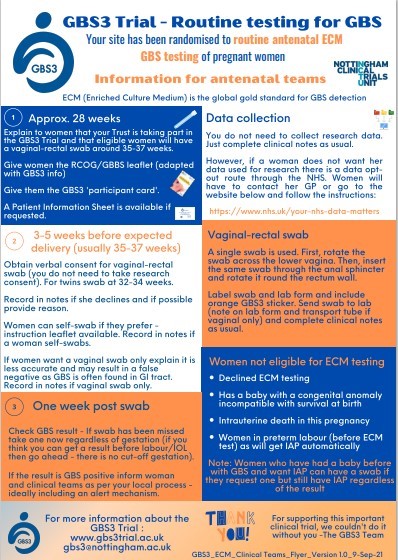


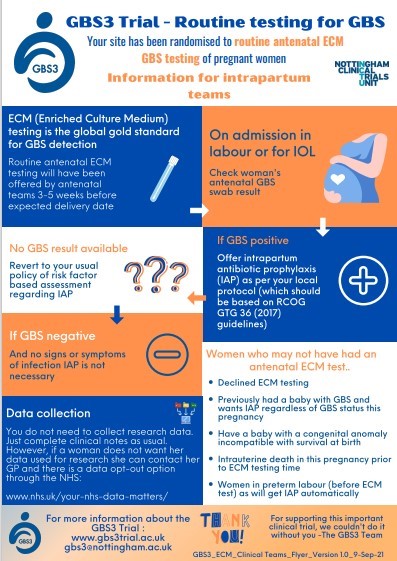


2b. Rapid testing protocol


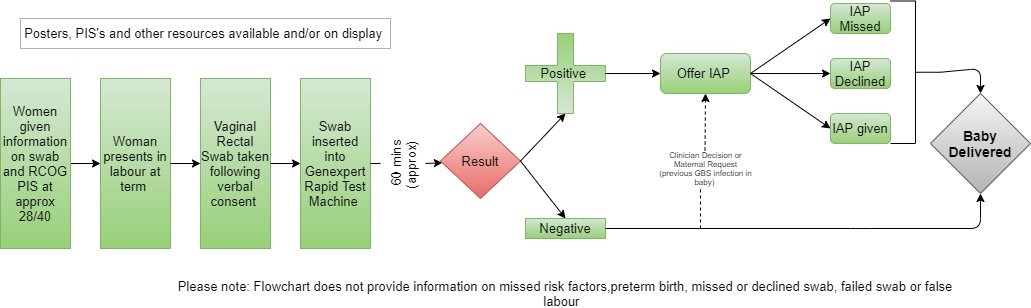


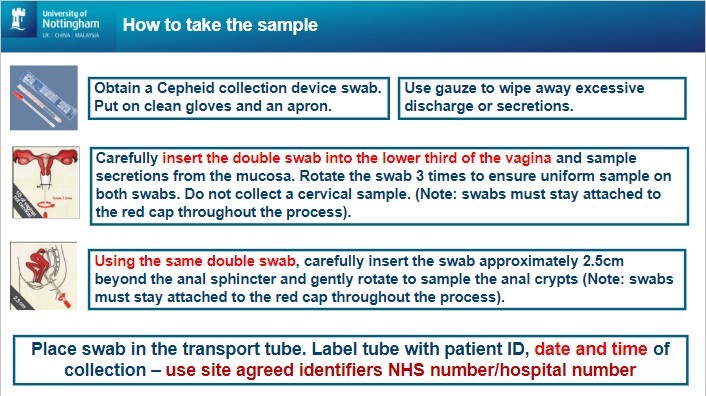


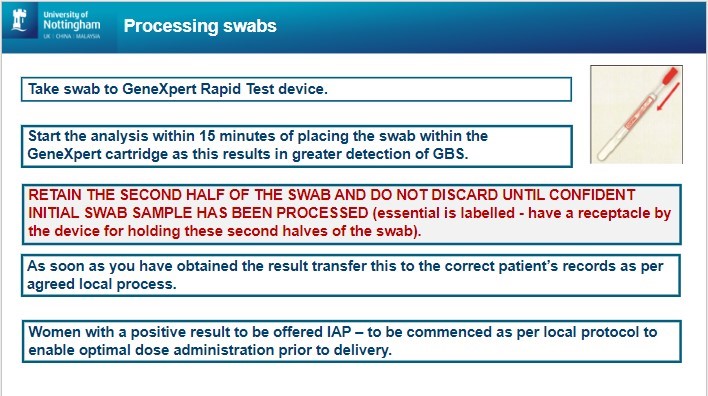

Supplement: online supplemental file 2 [file bmjopen-15-6-s002.docx]
