# Supplementary material for: Routine testing for group B streptococcus in pregnancy: protocol for a UK cluster randomised trial (GBS3)
Source: BMJ Open. 2025 Jun 17;15(6):e087887. doi: 10.1136/bmjopen-2024-087887 (PMC12182030; doi:10.1136/bmjopen-2024-087887)

Blinded Endpoint Adjudication Protocol: Accuracy of reporting of the primary outcome, clinically suspected all-cause early onset neonatal sepsis in the GBS3 trial

Version 2.0 23rd January 2024

Short Title of Trial: Routine Testing for Group B Streptococcus

Trial Registration: ISRCTN49639731

IRAS Project ID: 263682

Trial Sponsor: University of Nottingham

Sponsor Reference: 19056

Funder: NIHR Health Technology Assessment (17/86/06)

Key contacts

Name	Trial Role	Contact email
Professor Kate Walker	Deputy Chief Investigator	Kate.walker@nottingham.ac.uk
Professor Jane Daniels	Chief Investigator	Jane.daniels@nottingham.ac.uk
Dr Shalini Ojha	Co-investigator, Neonatologist	Shalini.ojha2@nottingham.ac.uk
Professor Jon Dorling	Co-investigator, Neonatologist	Jon.Dorling@uhs.nhs.uk
Kerry Barker-Williams	Lead Research Midwife	GBS3@nottingham.ac.uk
Dr Joanne Brooks	Trial Manager	GBS3@nottingham.ac.uk

Table of Contents

1. **Trial overview** 3
2. **Introduction** 4
3. **Aims of the adjudication process** 4
   Intrapartum stillbirths 5
4. **Methods** 5
   Number of cases to be adjudicated 6
   Site processes and data collection for EOS adjudication 6
5. **Central adjudication for definitive case ascertainment** 7
   Adjudicator panel composition 7
   Frequency of adjudication 7
   Method of adjudication 7
   Algorithm determination of clinically suspected EOS 8
   Analysis of clinically suspected EOS 8
   Intrapartum still birth 8
   Data Protection, Security and Confidentiality 8
6. **Updates to the Blinded Endpoint Adjudication Committee Protocol** 9
7. **Signature Page** 9
8. **References** 10
9. **Appendices** 11
   Appendix 1: Example Baby blood culture list 11
   Appendix 2: Flowchart 13

1. Trial overview

The GBS3 trial is a two arm, cluster randomised trial to determine the clinical and cost-effectiveness of testing for Group B Streptococcus in late pregnancy in the UK. The objective of the trial is to determine whether routine testing of women for GBS colonisation either in late pregnancy or during labour reduces the occurrence of all-cause early-onset neonatal sepsis, compared to the current risk factor- based strategy. It will involve approximately 320,000 women from up to 71 maternity units in England and Wales.

The routine testing strategies will use either antenatal Enriched Culture Medium (ECM) testing or intrapartum rapid testing using the Cepheid GeneXpert system (according to site randomisation), with Intrapartum Antibiotic Prophylaxis (IAP) offered if the test is positive for GBS presence in the sample taken. The control strategy is to offer IAP if a maternal risk factor for early-onset Group B Streptococcus (EOGBS) infection in her baby is identified before or arises during labour.

2. Introduction

The primary outcome for the GBS3 trial is all-cause early neonatal sepsis defined as:

- A positive culture of a pathogenic bacteria from blood or cerebrospinal fluid taken at <7 days of birth, **or**
- Death <7 days if infection or sepsis was recorded on the death certificate, **or**
- Negative/unknown culture status with ≥ 3 agreed clinical signs or symptoms , for which intravenous antibiotics are given for ≥ 5 days, starting within 7 days of birth..

Note: If the infant was discharged, or transferred prior to the completion of 5 days of intravenous antibiotics, the infant would still be classed as having sepsis if the intention was to treat for 5 or more days.

Data will be obtained from a variety of sources:

- UK Health Security Agency, Health Protection Wales
- Badgernet (Maternity and Neonatal)
- National Neonatal Research Database (NNRD)
- Paediatric Intensive Care Audit Network Database (PICANet)
- English Maternity Services Dataset, Welsh Maternity Indicators
- Hospital Episode Statistics (HES)

Clinically suspected cases of all-cause early onset sepsis (EOS) in babies born within trial sites will be detected using an algorithm that will use specific data fields and selected records for babies that meet the parameters that define a case. These data may be derived from the NNRD for babies admitted to the neonatal unit, and from the BadgerNet Maternity data, for babies remaining on the postnatal ward in selected sites. Central adjudication on a sample of cases of potential clinically suspected sepsis will ensure that the algorithm is robust.

3. Aims of the adjudication process

1. To create a standardised, centralised algorithm that identifies clinically suspected all-cause EOS from the GBS3 trial datasets.
2. To determine the diagnostic accuracy of the algorithm in ascertaining clinically suspected all-cause EOS against a reference standard of adjudicated cases.
3. To estimate the impact of missing routine data in case ascertainment, by estimating the false negative rate.
4. To compare the diagnostic accuracy between the two randomised allocation groups, to determine whether the routine data is differentially ascertaining clinically suspected EOS.
5. To monitor the prevalence of clinically suspected all-cause EOS between the two allocation groups, using the adjudicated cases.
6. To review individual level data of babies who die during labour to determine whether sepsis was the primary cause of death.

The DMC will consider the comparisons of measures of diagnostic accuracy (e.g. sensitivity, specificity, PPV, NPV) between randomised allocation groups, and make recommendations to the TMG, who would only be able to review pooled values.

Table 1: 2x2 table of diagnosis from algorithm and adjudication

		**Adjudication consensus**		
		Case	Not case	
**Algorithm used on data**	Case	TP	FP	PPV = TP/ (TP+FP)
	Not case	FN	TN	NPV = TN/ (TN+FN)
		Sn= TP/ (TP+FN)	Sp = TN/ (TN+FP)	
		FN rate = FN/ (TP+FN)	FP rate = FP/ (TN+FP)	

The above table considers the potential scenarios when adjudicating clinically suspected all cause EOS.

False positives are unlikely, as even if the clinical signs or symptoms that flagged the case in an algorithm are transient, the assumption will be that the antibiotics are working. However, they could inflate the event rate, so comparison of the PPV between groups is required, and should be independent of the prevalence of clinically suspected EOS.

False negatives will arise only if there is an inherent problem with the algorithm, or there is missing data: the latter is quite possible, will reduce the event rate and potentially introduce bias in the estimation of the effect of routine testing if differential at routine testing and risk factor sites.

The risk of differential ascertainment between trial sites is real: knowledge of maternal GBS colonisation in the testing sites could lead to more thorough review of the baby, longer hospital stays and potentially increased initiation of antibiotics. There will be a small risk that the mother was a false negative for the GBS test and was discharged early but any infection in the baby would probably be picked up as an emergency readmission.

Intrapartum stillbirths

All cases of babies who have died during labour or birth will be reviewed by the adjudication panel. The adjudicators will consider whether the primary cause of death is attributable to sepsis (either from post-mortem findings or positive microbiological results). If the primary cause of death is attributable to sepsis it will count towards the primary outcome. All cases of intrapartum stillbirths will be reported by participating sites during the period of data collection.

4. Methods

We will carry out a retrospective accuracy study of an algorithm to identify clinically suspected all-cause EOS cases, compared with the adjudicators consensus.

Number of cases to be adjudicated

The sample size for the GBS3 trial is 320,000 women and assumes a culture positive all-cause EOS rate of 0.98/1000 live births, equivalent to about 313 cases. The National Screening Committee model [1] considered a cohort of 711,999 live births, excluding elective Caesarean deliveries and estimated 351 cases of EOGBS infection across all gestation age deliveries under a risk factor based IAP strategy, giving rise to a rate of 0.0493%. Adding antenatal testing to the risk factor strategy was estimated to result in 294-299 EOGBS infections, a relative risk ratio of 0.84. This control rate is also between other estimates.[2, 3] Assuming GBS contributes 50% of all early-onset neonatal infection (the remainder *E. coli* (18%), other gram positives (23%),[4]) then the all-cause rate would be 0.98/1000 live births. The ratio of culture positive to clinically suspected EOS is estimated at 1:6 [5]. Given that we would expect a 40% reduction in the rate of all-cause EOS in the testing sites of the trial, we would expect a total of around 250 cases of culture positive all-cause EOS and 1500 cases of clinically-suspected EOS, so a total of approximately 1800 cases for our primary outcome. These would be distributed 1125 in the risk factor group and 675 in the testing group.

We plan to initially centrally adjudicate a sample of 300 potential cases of clinically suspected all-cause EOS, selected from 20 participating sites (average of 15 cases per site).

All cases of babies who have died during labour or birth, will be reviewed by the adjudication panel.

Site processes and data collection for EOS adjudication

71 sites in England and Wales are participating in the GBS3 trial. To ensure that the cases adjudicated are as representative as possible, 20 participating sites (10 testing sites, 10 risk factor sites) will identify cases of potential clinically suspected all-cause early onset neonatal sepsis. The 20 sites will be selected by the Nottingham Clinical Trials Unit (NCTU) from the GBS3 participating sites.

Each site will obtain a list of approximately 100 babies who have had a blood culture taken at less than 7 days after birth and the blood culture was negative or indeterminate during the routine data collection period from the local microbiology laboratory. All positive cultures are excluded from the adjudication process, as these automatically meet the primary outcome definition.

Of the babies who have had a negative or indeterminate blood culture result, the site will need to create an ordered list of babies which will be kept at site and report the number of potential clinically suspected EOS cases to the NCTU.

The ordered list will be prepared by an appropriate member of the site team (e.g. Consultant Microbiologist, Research midwife, Laboratory Technician etc).

The NCTU will provide the site with a list of 10 positions to be matched to the babies within the ordered list. See example in Appendix. From the remaining babies not selected, sites will select the first 5 babies that had ≥ 5 days of intravenous antibiotics starting within 7 days of birth. This activity will take place on one occasion during the routine data collection period at site.

The local research midwife will obtain the maternal and neonatal records for the 15 selected babies, copy (scan documents, take screenshots/create PDFs of the records) and the redact the identifying information and upload directly into the REDCap adjudication database. The NHS number, DOB and

postcode will be used as the record ID. REDCap will alert the NCTU when a new record is uploaded, NCTU will check that the record is properly redacted, including any site identifiers. This will be performed in accordance with the current version of the trial data management plan. The NHS number, DOB and postcode will not be provided to the independent adjudicators but stored separately to ensure data can be linked with routine data.

Site processes and data collection for stillbirth adjudication

Once all post-mortem investigations have been completed the local research midwife will copy (scan documents, take screenshots/ create PDFs of the records) and the redact the identifying information and upload directly into the REDCap adjudication database. The NHS number, DOB and postcode will be used as the record ID. REDCap will alert the NCTU when a new record is uploaded, NCTU will check that the record is properly redacted, including any site identifiers. This will be performed in accordance with the current version of the trial data management plan. The NHS number, DOB and postcode will not be provided to the independent adjudicators but stored separately to ensure data can be linked with routine data.

The number of cases reviewed by the obstetricians cannot be determined *a priori.*

5. Central adjudication for definitive case ascertainment

Adjudicator panel composition

Consultant neonatologists will be invited to act as adjudicators.

Two consultant neonatologists will look at each case independently. They will be asked to classify each case as primary outcome sepsis or non-sepsis. If they agree, then that case will be considered adjudicated. If they disagree, a third consultant neonatologist review the case. Each consultant neonatologist will be asked to review approximately 30 cases from sites other than their own.

Frequency of adjudication

Sites will undertake these activities at an appropriate time during the data collection period. Central adjudication will then be performed on an ongoing basis when the data is received from each site. The adjudicated outcome will be compared on receipt of the routine data and will be interrogated using the algorithm.

Method of adjudication

Members of the committee will review all available information and categorise potential cases into EOS or not, using the case definition of the actual duration of antibiotics of ≥ 5 days (or intended duration e.g. If the infant died, was discharged, or transferred prior to completion of 5 days of antibiotics) and ≥ 3 clinical signs or symptoms, within the first 7 days of life. Committee members will be asked to indicate the presence/absence of each of the clinical signs or symptoms listed in the definition of clinically suspected EOS.
The committee will be blind to the location of the baby and the GBS3 site allocation.

Members of the adjudication committee will use a REDCap form to adjudicate potential cases. The consensus diagnosis will form part of the GBS3 analysis dataset.

The opinion of the central adjudicators (after resolution of discrepancies as described above) will be used in reporting the trial results.

Algorithm determination of clinically suspected EOS

The 300 babies selected from the blood culture lists will be identified in the routine data and all the data fields pertaining to the case definition of clinically suspected EOS will be extracted. Calculated fields will determine intended (if baby dies or is transferred prior to completion of the 5 days) and actual duration of antibiotic use. The number of clinical signs and symptoms reported will be calculated. Infants where the algorithm identifies actual duration of antibiotics as ≥ 5 days and where ≥ 3 clinical signs are present will be classed as clinically suspected EOS cases, where these definitions are not met, this will be classed as not a case.

Missing data is likely to be an issue and will be present in the routine data as null fields, rather than coded as data unavailable. Thus, an algorithm selecting on presence of data will determine missing data as ‘symptom not present’.

Analysis of clinically suspected EOS

The number and percentage of the potential cases with clinically suspected EOS for both the adjudication consensus and the outcome derived from the routine sources will be presented overall and in each allocated group.

To evaluate the diagnostic accuracy of the algorithm in ascertaining clinically suspected all-cause EOS, a 2x2 table will be constructed for each allocation group to show the adjudication consensus against the outcome derived from the routine data sources. For each allocation group, summaries of diagnostic accuracy will be estimated with 95% confidence intervals.

This analysis will be conducted by an independent statistician and the results reported to the independent members of the Data Monitoring Committee.

Intrapartum still birth

Two obstetricians will look at each case independently. They will be asked to classify each case according to whether they consider the primary cause of death as sepsis or non-sepsis and record their decision on the REDCap database. If the two reviewers agree, then that case will be considered adjudicated. If they disagree, a third consultant obstetrician will review the case and their decision will be the outcome of the adjudicated case.

Data Protection, Security and Confidentiality

The information collected will be stored and handled in accordance with the data protection, security and confidentiality processes detailed in the current version of the GBS3 trial protocol.

6. Updates to the Blinded Endpoint Adjudication Committee Protocol

This plan will be reviewed and updated as required. Updates may be made for a number of reasons including protocol, substantial amendments, updates to documents and process changes.

Version number	Details/reason for change	Date approved/issued
Version 1.0	Version 1.0 of the Adjudication Protocol, there are no changes to be reported	23 Jul 2021
Version 1.1	Updates to flow diagrams and process of adjudication. Early onset sepsis definition updated in line with the trial protocol.	23 March 2023
Version 2.0	Updates to process of records selection. Updates to flow diagrams.	

7. Signature Page

Reviewed and approved by:

**Name**	Jane Daniels
**Role**	Chief Investigator
**Signature**	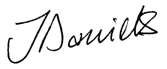
**Date (dd-mmm-yyyy)**	Jan 26, 2024

**Name**	Kate Walker
**Role**	Deputy Chief Investigator
**Signature**	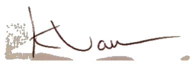
**Date (dd-mmm-yyyy)**	Jan 29, 2024

8. References

1. Bevan, D., et al., *Modelling the effect of the introduction of antenatal screening for group B Streptococcus (GBS) carriage in the UK.* BMJ Open, 2019. **9**(3): p. e024324.
2. O'Sullivan, C., et al., *Group B Streptococcal (GBS) disease in the UK and Irish infants younger than 90 days.* Arch Dis Child Fetal Neonatal Ed, 2016. **101**(A2): p. P3.
3. *Voluntary surveillance of pyogenic and non-pyogenic streptococcal bacteraemia in England, Wales and Northern Ireland*, in *Public Health England*. 2014, Public Health England: London.
4. Vergnano, S., et al., *Neonatal infections in England: the NeonIN surveillance network.* Archives of Disease in Childhood-Fetal and Neonatal Edition, 2011. **96**(1): p. F9-F14.
5. Klingenberg, C., et al., *Culture-Negative Early-Onset Neonatal Sepsis - At the Crossroad Between Efficient Sepsis Care and Antimicrobial Stewardship.* Frontiers in Pediatrics, 2018. **6**.

9. Appendices

Appendix 1: Example Baby blood culture list

If there were 32 babies who had a negative blood culture result, 10 would be selected from the list according to this schema:

List Order	NHS number (example)	.List Order	NCTU instruction
1	6940640	1	Ignore
2	2318718	2	Ignore
3	5024035	3	Ignore
4	5030506	4	Ignore
5	9353947	5	Select
6	9920006	6	Ignore
7	9639780	7	Ignore
8	3574238	8	Ignore
9	2466904	9	Ignore
10	1016722	10	Ignore
11	5252468	11	Ignore
12	3419271	12	Select
13	9011651	13	Ignore
14	1456642	14	Ignore
15	3593740	15	Select
16	8635688	16	Ignore
17	4168810	17	Select
18	1515038	18	Ignore
19	5534727	19	Ignore
20	1314614	20	Select
21	6260205	21	Ignore

22	1845984	22	Ignore
23	5742188	23	Ignore
24	9350316	24	Select
25	3678609	25	Ignore
26	3002624	26	Select
27	3487589	27	Select
28	1953400	28	Select
29	8923899	29	Select
30	3706217	30	Ignore
31	8593893	31	Ignore
32	3849200	32	Ignore

Appendix 2: Flowchart

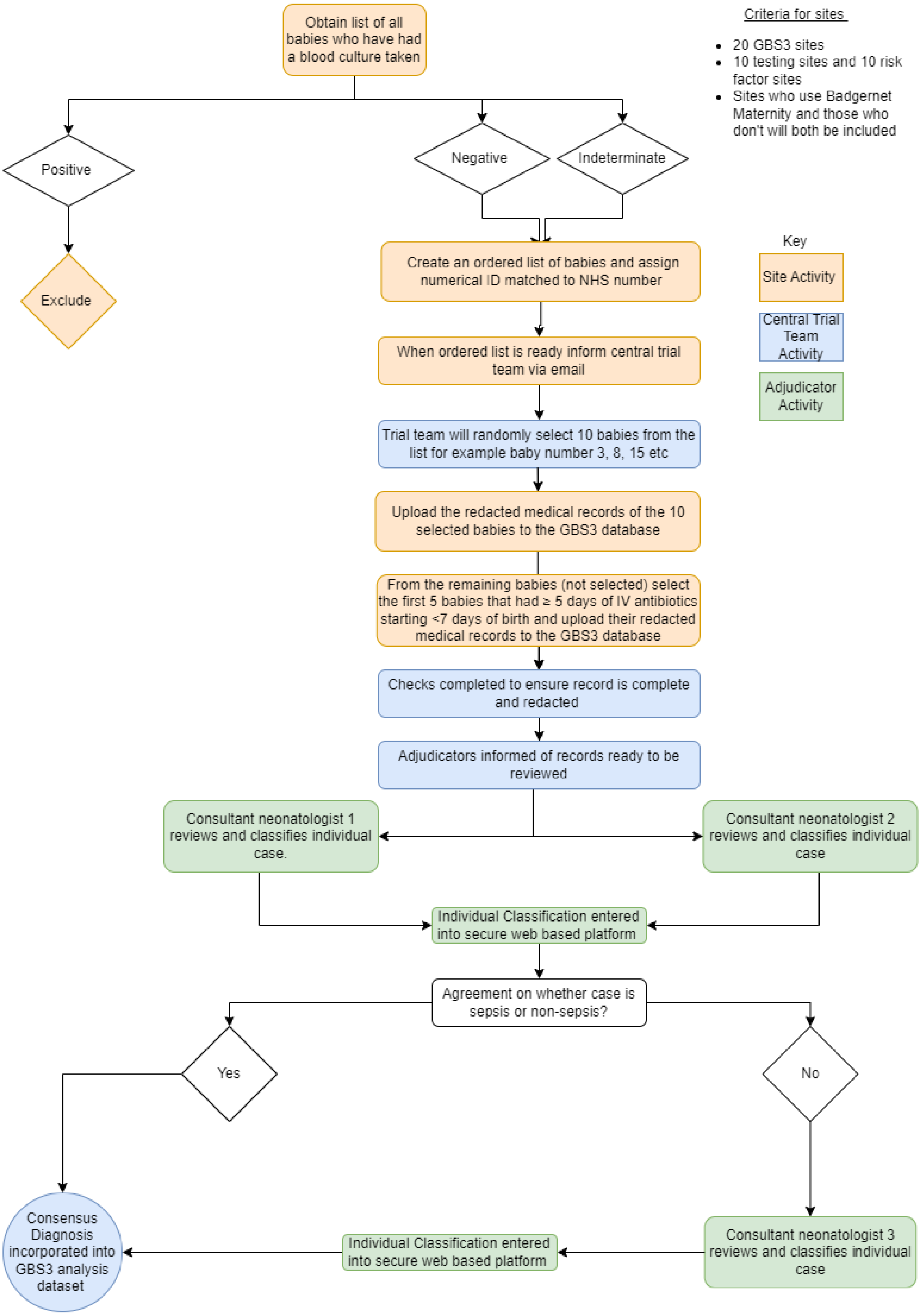

Supplement: online supplemental file 3 [file bmjopen-15-6-s003.pdf]
