# Supplementary material for: Routine testing for group B streptococcus in pregnancy: protocol for a UK cluster randomised trial (GBS3)
Source: BMJ Open. 2025 Jun 17;15(6):e087887. doi: 10.1136/bmjopen-2024-087887 (PMC12182030; doi:10.1136/bmjopen-2024-087887)

##

## Appendix 4. Data Flow Flowcharts

## 4a. Data flow charts ENGLAND


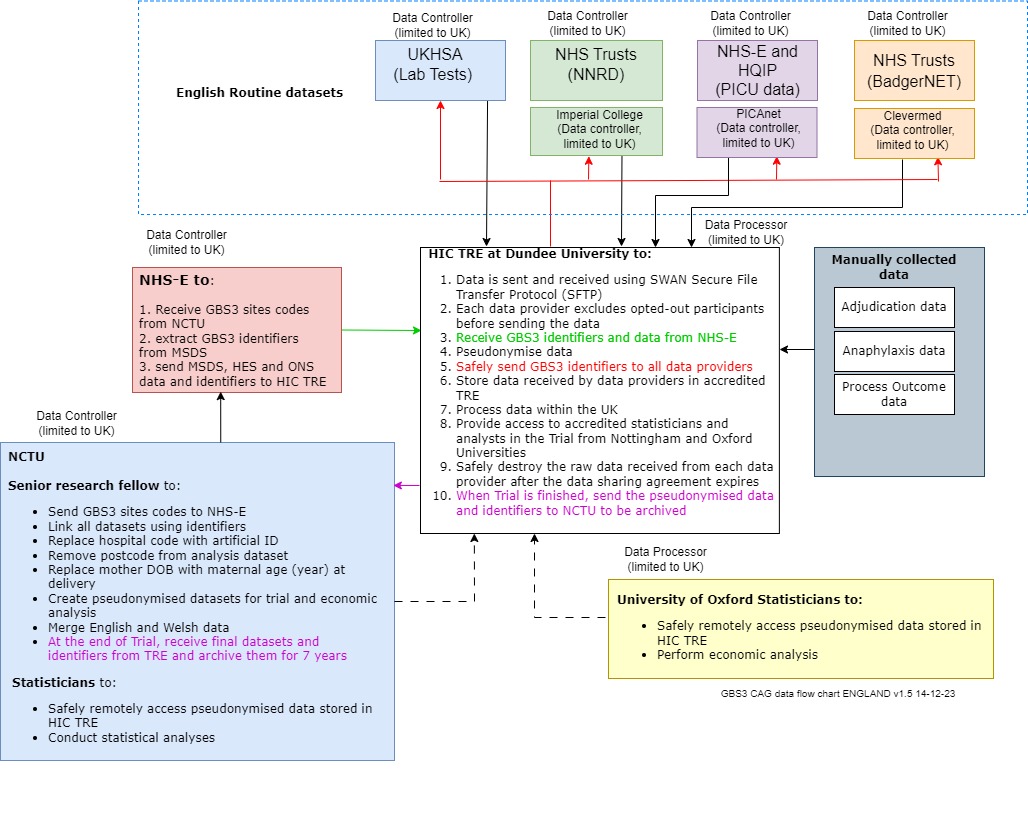


## 4b. Data flow charts WALES


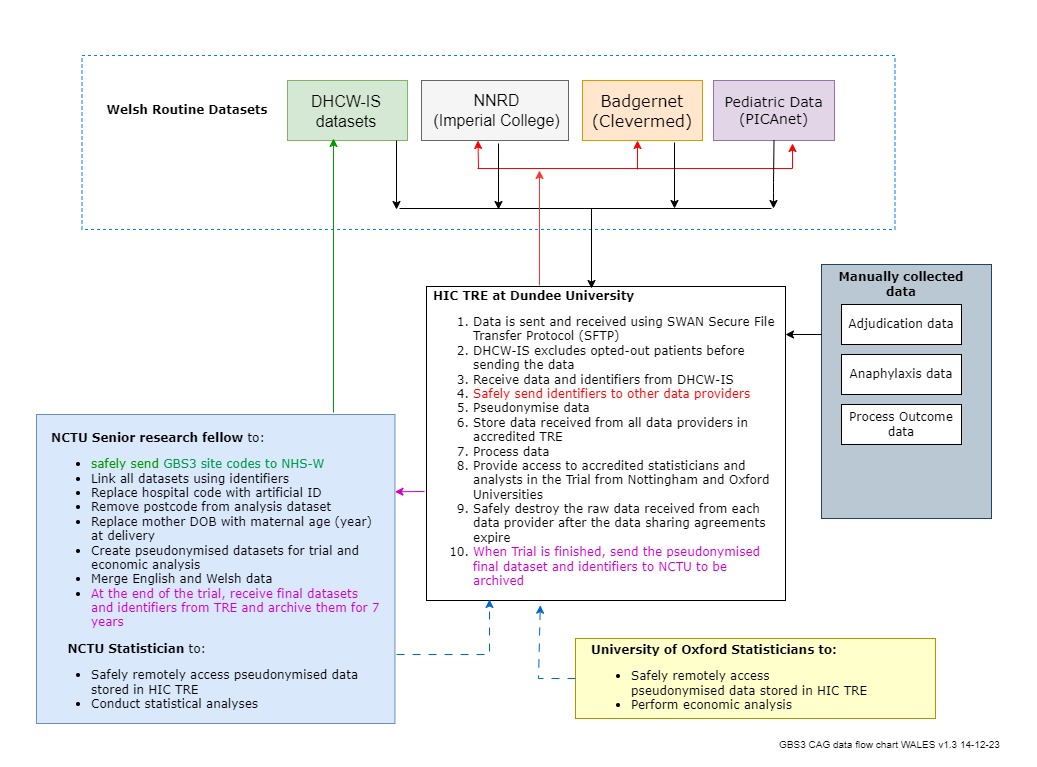


## 4c. Outcomes and Data Sources flowchart


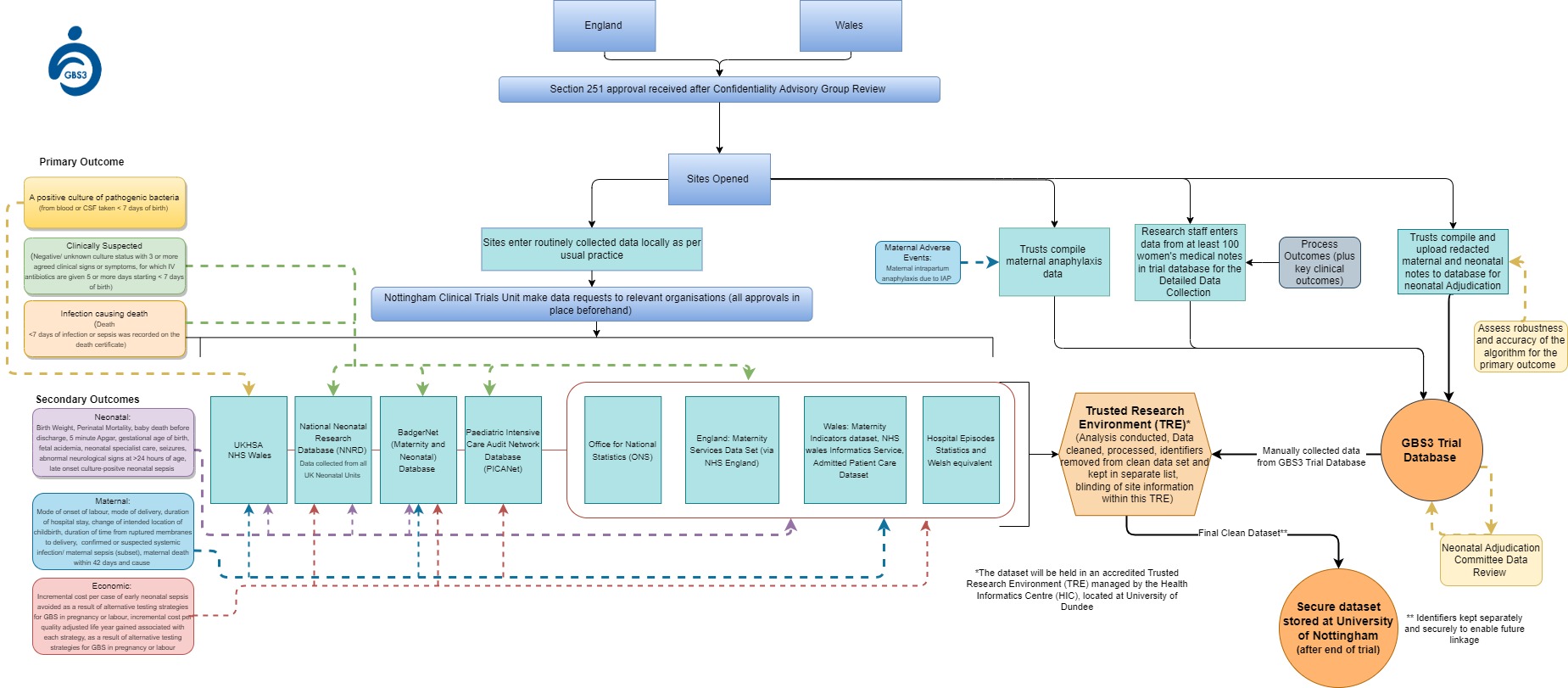

Supplement: online supplemental file 4 [file bmjopen-15-6-s004.docx]
