# Supplementary material for: Routine testing for group B streptococcus in pregnancy: protocol for a UK cluster randomised trial (GBS3)
Source: BMJ Open. 2025 Jun 17;15(6):e087887. doi: 10.1136/bmjopen-2024-087887 (PMC12182030; doi:10.1136/bmjopen-2024-087887)
Supplement: Supplementary file 5 [file bmjopen-2024-087887.reviewer_comments.pdf]

PEER REVIEW HISTORY

BMJ Open publishes all reviews undertaken for accepted manuscripts. Reviewers are asked to complete a checklist review form and are provided with free text boxes to elaborate on their assessment. These free text comments are reproduced below.

ARTICLE DETAILS

Title (Provisional)

ROUTINE TESTING FOR GROUP B STREPTOCOCCUS IN PREGNANCY: PROTOCOL FOR A UK CLUSTER RANDOMISED TRIAL (GBS3)

Authors

Daniels, Jane; Walker, Kate; Bradshaw, Lucy; Dorling, Jon; Ojha, Shalini; Gray, James; Thornton, Jim; Plumb, Jane; Petrou, Stavros; Madan, Jason; Achana, Felix; Ayers, Susan; Constantinou, Georgie; Mitchell, Eleanor J; Downe, Soo; Grace, Nicola; Plachcinski, Rachel; Cooper, Tracey; Moore, Sarah; Jones, Anne-Marie; Harrison, Eleanor; Brooks, Joanne; Barker-Williams, Kerry Barker-Williams; Hollands, Heidi; Mcleavey, Sarah-Kate; Willson, Seren; Webster, Sophie; Carpenter, Jodi; Hyslop-Peart, Meg; Wills, Louise; Haines, Rachel; Haydock, Rebecca; Sadiq, Shabina; Fiaschi, Linda; Evans, Lisa; Ogollah, Reuben; Seale, Jay; Spas, Susanne; Huang, Lixiao; Moody, Sebastian; Abramson, Janine; GBS3, Collaborative Group

---

VERSION 1 - REVIEW

**Reviewer**	**1**
**Name**	**Koliwer-Brandl, Hendrik**
**Affiliation**	**University of Zürich, Institute of Medical Microbiology**
**Date**	**03-Jun-2024**
**COI**	**I declare to have no competing interests.**

Thank you for giving me the opportunity to review your study protocol and I am looking forward to see the results of the study. In my opinion this clinical trial will be very important to further reduce the risk of EOGBS infection and even more to further reduce the risk of neonatal death. The protocol is well written and an important source of information for all interested in this topic, but also how a study protocol needs to be written. It is obvious that already a lot of persons are involved in writing this protocol and have edited it in its creation phase. Nevertheless, I have some small recommendation on the microbiological-diagnostic part of the protocol, as summarized below.

I miss some details about GBS detection using ECM or Xpert GBS:

1) Which enriched broth medium is required for the different labs? It would be good to standardize the procedure in the different laboratories.

2) After incubation in enriched culture broth medium a chromogenic agar plate is streaked out again from the broth? Page 66, line 35-37

2a) Which chromogenic agar is defined?

2b) How long is incubation of the chromogenic agar plate?

2c) Finally, how is GBS be identified in the different laboratories. MALDI-tof-MS identification is the current gold-standard in a diagnostic laboratory.

3) Cepheid released a new version of Xpert GBS having a higher sensitivity as compared to a previous version. It would be good to standardize all contributing labs use the same version of this PCR.

4) Please provide key statistics on the different assays, meaning sensitivities and specificities of rapid Xpert GBS and ECM test. Although, there is no direct comparison between both assays in each group, it would be helpful to compare the factors independently. It is still not clear in the field whether PCR has a comparable sensitivity to culture.

The reviewer provided a marked copy with additional comments. Please contact the publisher for full details.

**Reviewer**	**2**
**Name**	**Khalil, Mohammed Rohi**
**Affiliation**	**University of Southern Denmark**
**Date**	**21-Jun-2024**
**COI**	**No conflicting or competing interests**

The protocol titled "Routine Testing for Group B Streptococcus in Pregnancy: A Cluster Randomised Trial" suggests a study designed to evaluate the effectiveness of routine screening for Group B Streptococcus (GBS) in pregnant women. This protocol aims to provide robust evidence on the benefits and potential drawbacks of routine GBS testing in pregnancy, ultimately guiding clinical and policy decisions to improve maternal and neonatal health outcomes.

**Reviewer**	**3**
**Name**	**Fielding, Katherine**
**Affiliation**	**London School of Hygiene & Tropical Medicine**
**Date**	**24-Feb-2025**

COI **None**

Clear protocol for a cluster randomised trial, SPIRIT checklist used.

I have a few minor comments:

Methods & Analysis

1. In the exclusion criteria section, it is unclear if these are applied consistently across all three arms. More generally is the same population eligible for the ITT analysis population across all three arms?

Outcomes:

2. Primary outcome: what percentage of outcomes are reviewed by the adjudication panel? Why not all?

3. Secondary outcomes: there is a long list – can these be grouped into secondary and tertiary?

The process evaluation is a very important part of the study – data collected on at least 100 women/cluster. Data are collected retrospectively from the health records by the research midwife.

4. Are data sources are of good enough quality to obtain the listed process evaluation indicators?

5. I would recommend the authors use a process evaluation framework.

Sample size and justification:

6. please state the statistical package/command used for the sample size calculation.

7. The sample size calculation has been revised- add more detail of new assumptions (cluster size - coefficient of variation for cluster size and average cluster size).

Statistical analysis:

8. Who will be responsible for the independent review of the statistical analysis plan (DSMB?).

9. Logistic regression with random effects (clinic and women) used - explain how effect estimates of adjusted risk ratio and adjusted risk difference are calculated. What data on missing outcomes are you anticipating? When will multiple imputation be used?

10. A secondary analysis for the primary outcome is listed – sites excluded if “at least 80% uptake” not achieved - show is this defined in each study arm?

Qualitative evaluation:

11. What is the sample size

Ethics and dissemination

12. I assume the Ethics committees have given a waiver of consent for researchers to access their data? Please clarify

13. (I think) the acronym TMG is not defined in the article

VERSION 1 - AUTHOR RESPONSE

Reviewer: 1

1.1	Which enriched broth medium is required for the different labs? It would be good to standardize the procedure in the different laboratories.	The protocol required sites randomised to ECM to implement the 2018 Public Health England (PHE) Standards for Microbiological Investigation B58 issue 3.1 for Detection of Carriage of GBS, which is reference 18. This details the materials and process to follow by all sites randomised to ECM, and by all site when GBS testing was recommended by RCOG risk factor based guidelines.<br><br>As a microbiology standard, it would be inappropriate to selectively extract sections to respond to the reviewer's comments. A URL has been added to reference 18.<br><br>For reassurance to the reviewer, the enrichment broth is Lim broth
1.20	After incubation in enriched culture broth medium a chromogenic agar plate is streaked out again from the broth? Page 66, line 35-37	According to B58, the swab is incubated at 35-37°C in 5% CO_2 for 18-24hr in Lim broth and then plated onto solid medium.
1.21	Which chromogenic agar is defined? E.g. Granada plates	According to B58, the solid medium can be blood agar or selective agar or chromogenic agar.
1.22	How long is incubation of the chromogenic agar plate?	35-37°C in ambient air for 18-24hr
1.23	Finally, how is GBS be identified in the different laboratories. MALDI-tof-MS identification is the current gold-standard in a diagnostic laboratory.	B58 refers to a second PHE standard, ID4 Identification of Streptococcus species. We have added this as a reference along with a URL.<br>Audit of the ECM sites indicated MALDI-TOF was used for identification in all sites.

1.3	Cepheid released a new version of Xpert GBS having a higher sensitivity as compared to a previous version. It would be good to standardize all contributing labs use the same version of this PCR.	The Cepheid Xpert Xpress GBS was launched in the UK in September 2022, during the data collection phase of the trial. For practical, financial and analytical reasons, the first 10 rapid tests continued to use the original Xpert cartridges whilst the remaining 7 sites used the Xpress version from the start of their data collection period. Updates and improvements would occur in a real world situation, with hospitals switching to updated materials in a staggered fashion.<br>We discussed the Xpress Xpert with Cepheid before launch. The main improvements were the speed of obtaining a positive result, the inclusion of an internal control to indicate that sufficient sample is present and a second PCR target. We understand otherwise there is no difference, and the reported intrapartum sensitivity is 94% (95%CI 86-97%).
1.4	Please provide key statistics on the different assays, meaning sensitivities and specificities of rapid Xpert GBS and ECM test. Although, there is no direct comparison between both assays in each group, it would be helpful to compare the factors independently. It is still not clear in the field whether PCR has a comparable sensitivity to culture.	Can I refer the reviewer to Daniels J et al. A rapid intrapartum test for group B Streptococcus to reduce antibiotic usage in mothers with risk factors: the GBS2 cluster RCT. Health Technol Assess. 2022 Feb;26(12):1-82. doi: 10.3310/BICF1187. PMID: 35195519. This included a diagnostic accuracy study of intrapartum PCT (Cepheid Xpert GBS), performed by midwives, compared to intrapartum GBS, for accuracy statistics.<br>The reporting of accuracy parameters for antenatal microbiological testing is problematic because of the risk of bias and heterogeneity in the accuracy studies with respect to timing of test, samples taken (several report only on vaginal swabs) and culture method. A Cochrane review is planned,

		which would provide more appropriate statistics.<br>As a clarification, whilst the primary comparison is routine testing versus risk factor directed prophylaxis for early onset neonatal sepsis, there are secondary objectives to compare the two testing methods with respect to the uptake and test results being available in time to provide adequate antibiotic prophylaxis.

Reviewer: 2
No comments or questions

Reviewer: 3

#	Comment/ question	Response
3.1	Methods: In the exclusion criteria section, it is unclear if these are applied consistently across all three arms. More generally is the same population eligible for the ITT analysis population across all three arms?	The exclusion criteria for **testing** are not universal because of the nature of the risk for women electing or requiring an emergency Caesarean delivery and who are not in labour are not at risk. In ECM sites, these women would have received a test 3-5 weeks prior to delivery in case they went into spontaneous labour.<br>The **dataset** population is identical across the randomised groups (i.e. all sites).
3.2	Outcomes: Primary outcome: what percentage of outcomes are reviewed by the adjudication panel? Why not all?	We anticipated 1,800 clinically suspected cases, although more recent data suggest this could be higher. Adjudication requires collation of all maternal and neonatal notes and review by a consultant neonatologist, taking >1 hour per case with associated costs. Adjudication for all suspected cases was deemed not feasible, so a compromise of 300 cases was chosen and affordable. See Appendix 3.
3.3	Secondary outcomes: there is a long list – can these be grouped into secondary and tertiary?	It is important to consider both maternal and neonatal consequences of early onset sepsis, as well as capture key

		drivers of cost. Whilst a core outcome set was published after the design of the trial, the neonatal outcomes reflect that COS. The SAP has been approved by the DMC and analysis has commenced, so we are disinclined to redefine the hierarchy of outcomes.
3.4	The process evaluation is a very important part of the study – data collected on at least 100 women/cluster. Data are collected retrospectively from the health records by the research midwife.<br>Are data sources are of good enough quality to obtain the listed process evaluation indicators?	Patient records are the only source of the process outcomes. Data collection is now complete and we are satisfied with the completeness of data collection.
3.5	I would recommend the authors use a process evaluation framework.	We understand the question, and the UK MRC Process Evaluation for Complex Interventions (Moore 2015) would be the obvious choice, there are externalities that limit our ability to follow this approach. First, GBS3 was a commissioned project and a full implementation evaluation was not in the original brief and so not resourced. We have now published our qualitative study and we intend to do some quantitative evaluation of the predictors of neonatal outcomes ad testing rates. Second, all of the results will be used by the UK National Screening Programme and will be integral in the assessment against their principles for recommending a screening programme. Our economic evaluation will be extended to provide an impact assessment. Whilst linked, this is a separate piece of work and too much to covered in a BMJ Open protocol paper.
3.6	Sample size and justification please state the statistical package/command used for the sample size calculation.	For the original sample size calculation, we used PASS software to calculate the sample size without design effect adjustment based on Z-test for two proportions (Normal approximation) with the following

		parameters: two-sided alpha=0.05, group allocation 1:1, power=90%, p1= 0.000986, p2=0.0005916 giving a sample size of 212960.<br>We then inflated the sample size to account for design effect by multiplying the sample size by the design effect of 1.5 calculated from the formula below:<br>$Design\ effect = 1 + \{(1 + cv^2)\overline{m}\text{-}1\}\rho$<br>$Where,$<br>$cv \quad = coefficient\ of\ variation = 0.31$<br>$\overline{m} = cluster\ size = 4500$<br>$\rho = ICC = 0.0001$<br>We have added this detail in the manuscript.<br>**Stata calculator**<br>`Di 1+((1+0.31^2)*(4500-1))*0.0001`<br>`1.4931354 ~1.5`
3.7	The sample size calculation has been revised- add more detail of new assumptions (cluster size - coefficient of variation for cluster size and average cluster size).	The sample size was not changed however, there were a number internal and external factors that impacted on assumptions relating to cluster size during the trial. With the reduced number of births per year requirement for hospitals and variable data collection period, the coefficient of variation increased from 0.31 to 0.46 and average cluster size to 4750.
3.8	Statistical analysis: Who will be responsible for the independent review of the statistical analysis plan (DSMB?).	The statistical analysis plan was reviewed by both the Trial Steering Committee and Data Monitoring Committee
3.9	Logistic regression with random effects (clinic and women) used - explain how effect estimates of adjusted risk ratio and adjusted risk difference are calculated. What data on missing outcomes are you anticipating? When will multiple imputation be used?	The adjusted risk ratio and risk difference with corresponding 95% confidence intervals are obtained using Stata's margins command with standard errors computed using the delta method.<br>We have now appended the SAP with details of multiple imputation including the imputation model.
3.10	A secondary analysis for the primary outcome is listed – sites excluded if "at least 80% uptake"	For this analysis, the corresponding risk factor site

	not achieved - show is this defined in each study arm?	opened at the same time as the routine testing site who failed to reached 80% testing coverage will be excluded.<br>We've added this detail.
3.11	Qualitative evaluation: What is the sample size	We have inserted a statement on the anticipated number of interview participants for the qualitative study on page 18.
3.12	Ethics and dissemination I assume the Ethics committees have given a waiver of consent for researchers to access their data? Please clarify	We describe the absence of research consent on page 18. This was approved by the REC, but in the UK is not called a consent waiver.
3.13	(I think) the acronym TMG is not defined in the article	Thank you for spotting this acronym. This is now defined as the Trial Management Group on page 18.
